# A systematic review of intervention effects on potential mediators of children’s physical activity

**DOI:** 10.1186/1471-2458-13-165

**Published:** 2013-02-23

**Authors:** Helen Brown, Clare Hume, Natalie Pearson, Jo Salmon

**Affiliations:** 1 School of Exercise and Nutrition Sciences, Deakin University, Melbourne, Australia; 2 School of Sport, Exercise & Health Sciences, Loughborough University, Loughborough, UK

**Keywords:** Mediator, Child, Physical activity promotion, Theory

## Abstract

**Background:**

Many interventions aiming to increase children’s physical activity have been developed and implemented in a variety of settings, and these interventions have previously been reviewed; however the focus of these reviews tends to be on the intervention effects on physical activity outcomes without consideration of the reasons and pathways leading to intervention success or otherwise.

To systematically review the efficacy of physical activity interventions targeting 5-12 year old children on potential mediators and, where possible, to calculate the size of the intervention effect on the potential mediator.

**Methods:**

A systematic search identified intervention studies that reported outcomes on potential mediators of physical activity among 5-12 year old children. Original research articles published between 1985 and April 2012 were reviewed.

**Results:**

Eighteen potential mediators were identified from 31 studies. Positive effects on cognitive/psychological potential mediators were reported in 15 out of 31 studies. Positive effects on social environmental potential mediators were reported in three out of seven studies, and no effects on the physical environment were reported. Although no studies were identified that performed a mediating analysis, 33 positive intervention effects were found on targeted potential mediators (with effect sizes ranging from small to large) and 73% of the time a positive effect on the physical activity outcome was reported.

**Conclusions:**

Many studies have reported null intervention effects on potential mediators of children’s physical activity; however, it is important that intervention studies statistically examine the mediating effects of interventions so the most effective strategies can be implemented in future programs.

## Background

The physical, mental and social benefits of physical activity for children are widely acknowledged [1-5]. In spite of public health recommendations for children to spend at least 60 minutes each day in moderate- to vigorous-intensity physical activity [6-8], many children are not meeting the minimum recommended levels [9]. Of further concern, there is evidence of substantial declines in physical activity levels from childhood through to adolescence [10-14]. It is therefore important to address physical activity participation during childhood through the development of effective and efficacious intervention strategies.

Many interventions aiming to increase children’s physical activity have been developed and implemented in a variety of settings, and these interventions have previously been reviewed [15-17], with a recent review suggesting an overall lack of effectiveness of interventions to increase children’s objectively measured physical activity. The focus of these reviews tends to be on the intervention effects on physical activity outcomes; however, the reasons and pathways leading to intervention success or otherwise are also important to identify.

Kamath and colleagues performed a meta-analysis with 18 studies that aimed to promote physical activity among 2-18 year olds (studies published since each database’s inception to February 2006 were included) [18]. The meta-analysis reported a small but statistically significant pooled effect size of 0.12 (0.04, 0.20) on increases in physical activity. Interestingly, that review also reported stronger effects for studies that utilised multiple cognitive approaches (e.g., goal setting, problem solving/relapse prevention) and stronger effects for studies that included behavioural reinforcement. It has been argued that for the development of interventions that result in long-term behaviour change, understanding the mechanisms through which the intervention achieved success is critical [19].

The mechanisms by which intervention strategies achieve their effect are usually through intermediate or mediating variables that are hypothesised to be causally related to the outcome of interest. Mediators can be defined as “intervening causal variables that are necessary to complete a cause-effect pathway between an intervention and physical activity” [20]. Potential mediators are identified in behavioural theories such as social cognitive theory [21] and the theory of planned behaviour [22], and while studies often use these theoretical frameworks to guide their interventions the success of targeting these mediators is not well understood. It is suggested that formal mediating analysis is undertaken to determine the causal sequence between the intervention and the outcome by identifying if the independent variable (i.e. the intervention) exerts its effect on the outcome (i.e. physical activity) through a proposed mediating variable [19,23].

There are several approaches to establishing mediation, however the basic process involves testing how the independent variable changes the mediating variable (action theory), how the mediating variable influences the outcome controlling for the independent variable (conceptual theory) and the mediated effect test to explore the extent of the mediated effect on the intervention effect on the outcome [24].

The development of-theory based interventions identifying mediators of change in physical activity is highly complex, partly due to the often subjective nature of behavioural measures and associated measurement difficulties, particularly in children. Children present specific challenges for physical activity measurement due to the differing rates of maturation and development among children of the same age, their lower levels of cognitive functioning which affects their ability to think abstractly and perform detailed recall. Children also have a more sporadic and variable physical activity pattern than adults, making objective measurement more difficult [25,26].

This current review aims to develop and add to previous reviews of mediators of physical activity interventions in young people [27-29] by including all physical activity interventions that have reported on mediators, and by calculating effect sizes for intervention effects on mediators. Previous reviews are limited because they either excluded studies that did not report a mediating analysis [27,28] or relied on statistical significance only for determining intervention effects [17]. While no formal statistical mediation analyses were reported in previously reviewed studies, direct intervention effects on potential mediators (as indicated by tests of statistical significance) were variable and tended to be more successful if the intervention also positively impacted on children’s physical activity. Emphasis on the statistical significance alone, as relied on by Salmon and colleagues, may be inappropriate as a sufficiently large sample size may result in a statistically significant result for even trivial effects [30,31], conversely a small sample size may result in a lack of statistical power for detecting meaningful effects. Therefore calculating an effect size enables the investigator to interpret the magnitude of the intervention effect on potential mediators irrespective of sample size [32].

Furthermore, this review will focus on children aged 5-12 years because this covers the complex and dynamic time periods when physical activity levels begin to decline. Previous reviews have covered wider age groups (e.g. 2-18 years) [17] but have made little distinction between the intervention effects on younger versus older children.

Therefore, the aim of this paper was to provide an up-to-date review of the efficacy of physical activity interventions targeting 5-12 year old children on potential mediators and, where possible, to calculate the size of the intervention effect on the potential mediator. Intervention effects on the physical activity outcomes were also examined.

## Methods

**Figure 1:**
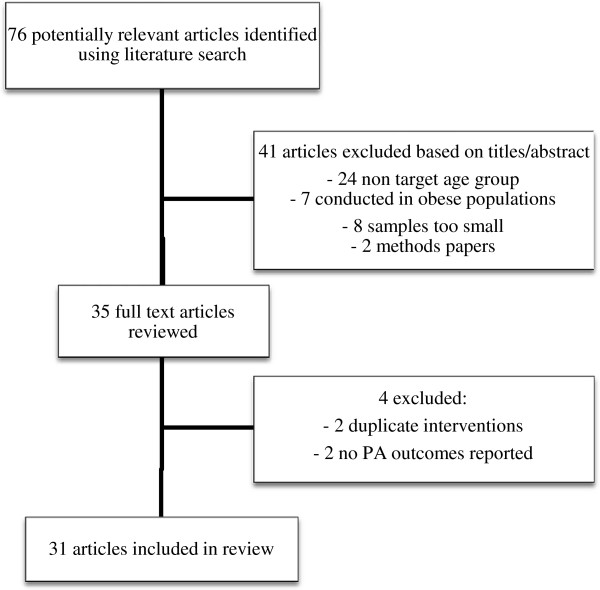
Flow of studies through the review process.

A comprehensive search of published studies was conducted using the computer databases Medline and Premedline; SCOPUS; Sport Discus; CINAHL; Science Direct; PsycARTICLES; PsycInfo; Cochrane, Social Scisearch and all Ovid databases. A search was conducted for articles in the English language published from 1985 to April 2012. Subject terms included physical activity, children, youth, mediation, mediator, intervention, randomiz(s)ed controlled trial. The flow of studies through the review process is reported in Figure 1.

To be eligible for inclusion in the current review, studies had to: 1) be a randomised controlled trial (RCT), group RCT, comparative or concurrent trial or quasi- experimental study; 2) have a sample that included elementary school aged children (5-12 years) at baseline; 3) have a sample size greater than 30; and 4) report potential mediators of physical activity change. Overweight or obesity treatment studies or studies of clinical populations were excluded. Article selection and data extraction was performed by the authors. When opinions differed over inclusion, consensus was reached through discussion.

### Data extraction

Data was extracted onto forms developed for this review. The following data were extracted: author, date and country of study, study design, description of the intervention (e.g. duration, theoretical underpinning, strategies used in the intervention), aims, characteristics of the participants (sample size, age, gender), physical activity outcome variables and measures, potential physical activity mediator variables and measures, intervention effects on outcome and potential mediating variables (refer Additional file 1: Table S1). Data were extracted by a co-author for a random selection of approximately 12 studies and were found to be consistently reported.

Where effect sizes of the intervention on potential mediators were not reported, the effect size for studies that reported a significant direct effect on the mediator was calculated. In studies where there is a comparison made between two groups (e.g. control vs intervention), effect sizes can be measured either as the standardised difference between the two group means *or* as the effect size correlation (*r*) which informs on the magnitude of the effect on participants of being assigned to either control or intervention groups [30,31]. Effect sizes were unable to be calculated for six studies due to insufficient information [33-38]. Effect sizes (Cohen’s *d*) for the intervention on potential mediators for the remaining studies were calculated by using formula described by Lipsey and Wilson [39]. In line with Cohen’s classification [40], effect size was defined at four levels: (≤0.2) trivial, (>0.2-0.5) small, (>0.5-0.8) moderate, and (>0.8) large.

### Coding intervention effects

To summarise the intervention effects on potential mediators of physical activity change, studies were grouped by potential mediator variables within levels of influence according to ecological models [41]: cognitive/psychological (e.g. knowledge, self-efficacy); social environment (e.g. family support for physical activity); and physical environment.

## Results

### Search results

Thirty-one intervention studies were eligible for inclusion in this review. Interventions were published between 1985 and April 2012 (See Additional file 1: Table S1). The majority of studies (n=21) were RCTs [33,37,38,42-59], five studies were quasi-experimental in design [36,60-63], four were comparative/concurrent trials [34,35,64,65], and one used a crossover design [66]. Twenty-three studies were conducted in the US, with most of the remainder of studies from Europe. Increasing physical activity was the primary aim in 15 studies [35-38,44,46-50,52,58,63,65-67]; decreasing cardiovascular disease risk factors was the primary aim in four studies [33,42,45,62]; general health promotion was the primary aim in two studies [34,51] and nine studies reported obesity prevention as the primary aim [43,53-57,59,60,68]. Two studies examined intervention effects on potential mediators separately for boys and girls [48,65].

### Theoretical frameworks

Social Cognitive Theory (SCT) was reported as the theoretical basis in ten studies [42,47,54-57,59,62,65,69] and Social Learning Theory in four studies [33,34,43,70]. Single studies reported using Theory of Planned Behaviour (TPB) [44] and the Social Ecological Model (SEM) [48]. Four studies reported aspects of two or more theoretical frameworks [35,42,65,68]; Harrell et al incorporated SCT and Theory of Planned Behaviour (TPB) [42], Gortmaker et al used SCT and Behavioural Choice Theory [68], Pate et al applied SCT and Pender’s Health Promotion Model (HPM) [65] and Jurg et al incorporated aspects of SEM, TPB, Physical Exercise and Habit, Precaution Adoption Process Model and Service Quality Model [36]. Twelve interventions did not describe a theoretical framework.

### Intervention effects on potential mediators

Table 1 provides a summary of the direction and size of association between the intervention and the potential mediators. Eighteen potential mediators were identified from the 31 studies that met the inclusion criteria, most of which were cognitive/psychological (n= 15) All studies used questionnaires to measure the mediating variables. None of the studies included in this review reported conducting formal mediating analysis.

**Table 1:** Summary of size and association between the intervention and potential mediator and physical activity outcomes

		**Studies with positive significant effect on potential mediator (and rating of effect size)**	Physical activity outcomes*	**Studies with no significant effect on Potential mediator (and rating of effect size)**	Physical activity outcomes*
**Cognitive/psychological**					
Self-efficacy towards PA	14	T [45,47,52]	+ [35,43,45,47,52,62]	T [49,53,59]	+ [49,53,59]
		S [43,63] (gp 1&2)		S [55]	
		L [62]	0 [63] (gp 1&2)	M [57]	0 [36,37,55,57]
		NC [35]		NC [36,37]	
Self-perception/self-esteem	5	L [63] (gp.1)	+ [63] (gp.1)	T [67]	+ [57,63] (gp 2), [67]
				S [55,57]	
				M [56]	0 [55,56]
				L [63] (gp 2)	
Knowledge	9	S [42,43]	+ [43,59,71]	S [66]	+ [66]
		M [59,68,71]		NC [37]	
		NC [33,34]	0 [33,34,42,68]		0 [37]
Enjoyment of PA	6	L [49,60]	+ [49,60]	T [48] (boys)	+ [48] (boys), [48] (girls),
				S [48] (girls), [56,67]	[53,67]
				M [53]	
					0 [56]
Intention to be physically active	5	T [65] (boys)	+ [46]	S [67]	+ [36,67]
		M [46]		M [65] (girls)	
			0 [65] (boys)	NC [36,38]	0 [38,65] (girls)
Outcome expectancies	5	S [47]	+ [36,47]	T [49]	+ [49,57]
		NC [36]		S [55,57]	0 [55]
Preference for PA	4	S [57]	+ [46,57]	S [55]	0 [54,55]
		L [46]		L [54]	
Attitude towards PA	2	M [44]	+ [44]	T [66]	0 [66]
Perception of safety	2	-	-	T [49,50]	+ [49]
					0 [50]
PA beliefs	2	S [65] (boys and girls)	0 [65] (boys and girls)	T [47]	+ [47]
Habit	1	NC [36]	+ [36]	-	
Attraction to PA	1	-	-	M [46]	+ [46]
Exercise behavioural capability	1	NC [35]	0 [35]	-	-
Awareness of PA levels	1	-	-	NC [36]	+ [36]
Perceived behavioural control	1	NC [38]	0 [38]	-	-
**Social Environmental**					
Social support for PA	6	T [47,65] (boys)	+ [47]	T [49]	+ [36,45,49,53]
				S [53]	
			0 [65] (boys)	M [65] (girls)	0 [65] (girls)
				L [45]	
				NC [36]	
Family support for PA	4	L [58] (mother)	0 [58] (mother)	T [58] (father)	+ [47,57,67]
				S [47,57,67]	0 [58] (father)
Peer support for PA	1	-	-	S [47]	+ [47]
**Physical environmental**	0	0		0	

KEY:

Effect on PA: + = significant effect on physical activity (PA) outcome, 0= no significant effect on PA outcome, +/0= mixed result.

Effect size: T= trivial (≤0.2), S= small (>0.2-0.5), M= moderate (>0.5-0.8), L= large (>0.8), NC=not able to be calculated.

### Cognitive/psychological potential mediators

Fifteen cognitive/psychological mediators were identified from the included intervention studies.

#### Self-efficacy

Self-efficacy was the most commonly examined potential mediator (n=14 studies). Seven studies found a positive significant effect on self-efficacy, with small to large effect sizes ranging from 0.11 to 0.82 [35,43,45,47,52,62,63]. A positive intervention effect on physical activity was found in six of these studies. Self-perception/self-esteem was also targeted in six studies, with small to large effect sizes ranging from 0.16 to 0.80 and a positive significant effect on this potential mediator found only in one study [63] (in one group only). A positive intervention effect on physical activity was also found in this study.

#### Knowledge

Knowledge was examined in nine studies [33,34,37,42,43,45,59,68,71]. Seven of these reported positive intervention effects [33,34,42,43,59,68,71], with six reporting small-moderate effects ranging from 0.34-0.69 [42,43,59,66,68,71]; the remaining two studies did not supply enough data to calculate the effect size. Four out of the nine studies reporting positive intervention effects on knowledge also reported positive intervention effects on the physical activity outcome [42,43,51,59].

#### Intention to be physically active

Intentions to be active were examined in five studies [36,46,61,65]. Positive intervention effects were found in two of these studies (one in boys only) [46,65], with trivial to moderate effect sizes. Of these two studies a positive significant effect on the physical activity outcome was reported in one [46].

#### Enjoyment of physical activity

Enjoyment of physical activity was examined in six studies [48,49,53,56,60,67]. Two studies found large intervention effects on children’s self-reported physical activity enjoyment, with effect sizes of 1.62 and 0.93. Both studies also showed positive intervention effects on physical activity [49,60].

#### Outcome expectancies

Outcome expectancies were examined in five studies [36,47,49,55,57]. Positive intervention effects were found in two of these studies, with a small effect size found for one of the studies (which also had mixed intervention effects on physical activity) [47]. The effect size of the other study was unable to be calculated due to lack of available data, however this intervention had a positive effect on physical activity [36].

#### Preference for physical activity

Preference for physical activity was examined in four studies [46,54,55,57]. Positive intervention effects as well as positive effects on the physical activity outcome were found in two of these studies [46,57].

#### Other cognitive/psychological potential mediators

Positive significant intervention effects on attitude were shown in one out of two studies [44]. This study also reported a positive effect on the physical activity outcome. A positive intervention effect was also found for habit in one study [36] and exercise behavioural capability in one study [35], however there was not enough data available to calculate the effect sizes. A positive significant intervention effect on PA beliefs were found in one out of two studies, however the intervention showed no effect on the physical activity outcome [65]. Awareness of physical activity levels was examined in just one study, and no intervention effects on that mediator were found [36]. No significant intervention effects were found for attraction, perception of safety and perceived behavioural control.

### Social environment potential mediators

Three social environmental potential mediators were identified from the included intervention studies.

#### Social support for physical activity

Social support for physical activity was the most commonly examined social environmental mediator (n=6 studies) [36,45,47,53,54,65]. Two studies found trivial effect sizes when targeting social support for physical activity, however one of the studies reported this result for boys only [65]. Five studies targeted family support for physical activity; with only one study reporting significant intervention effects on this potential mediator [58]. This study reported effects among mothers and fathers separately and only the results of mothers were found to have significant effects on family support. Peer social support was examined in one study [47]; however, there were no significant effects on this potential mediator.

### Perceived physical environment potential mediators

No studies examined physical environment potential mediators.

### Settings and strategies used

Twenty studies were based in the school setting [33-36,42-53,60,66-68]; eight in the family setting [37,38,54-59]; two in the afterschool setting [62,63] and a single study was based in the community setting [65]. As indicated in the supplementary table, a wide variety of strategies were used in the studies, including curriculum delivery, tailored physical education classes, environmental changes, activity class breaks, active transport campaigns, newsletters to families, family events, active homework, program delivery via the internet, self -management assistance and community linkages. Of the 31 studies in this review, 29 used a different combination of these strategies to deliver their intervention. While it would have been useful for this review to explore whether potential mediators were appropriately targeted and matched with strategies and also whether they were assessed at the appropriate time point using valid and reliable measures, unfortunately the methods used in studies were often not clearly described or lacked detail and as such, we are unable to comment on whether the conclusions drawn here would be different if such information were available.

## Discussion

Understanding the mechanisms through which interventions achieve success in changing the physical activity behaviours of children is imperative. The aim of this paper was to review evidence of the efficacy (and size of effect) of physical activity interventions targeting 5-12 year old children on potential physical activity mediators and to examine whether success in promoting physical activity varied in terms of potential mediator outcomes. Thirty-one intervention studies published between 1985 and April 2012 satisfied the criteria for inclusion in this review with 18 mediators identified and 77 outcomes on potential mediators reported (nb: these are not mutually exclusive as some studies targeted multiple mediators and reported results separately by sex). There were 33 positive intervention outcomes on the targeted potential mediators and 73% of the time a positive effect on physical activity was also reported. In contrast, where a null effect on a potential mediator was reported (44 results reviewed), a positive effect on children’s physical activity was identified on just 54% of occasions. Although none of these studies performed a mediating analysis, the results suggest that where a positive intervention effect on the mediator was found, there was more likely to be a positive effect on physical activity.

This review confirms that physical activity is a complex entity and that the potential mechanisms of change are multifactorial. Consistent with previous reviews [27-29], self-efficacy, knowledge, intentions, enjoyment, and social support were the most commonly targeted. The results of this review are presented according to an ecological framework and clearly show that much of the focus of previous children’s physical activity interventions has been on cognitive/psychological factors with very few studies targeting the broad range of social, physical, cultural or policy environmental influences, particularly not concurrently. The current review included a broader range of potential mediators for consideration than previous reviews that have only included studies that conducted statistical tests of mediation in adolescent and child interventions [27,72]. A more inclusive review of potential mediators was deemed important for informing the development of more effective strategies that could be incorporated into future interventions, and also for identifying the gaps in the types of potential mediators that should or could be targeted.

Given the target age of children in these interventions, it is somewhat surprising that the majority of intervention research has focused on cognitive/psychological aspects of children’s physical activity at an age where children’s autonomy is just emerging and the opportunity to be physically active is likely to be highly dependent on adult carers (i.e., parents, grandparents, teachers). Fewer than 40% of studies in the current review reported a positive impact on cognitive/psychological potential mediators. The most effective changes reported were in children’s knowledge of physical activity, which may not translate into change of behaviour [73,74]. While enjoyment has been found to be a significant correlate of children’s physical activity, other potential cognitive mediators targeted in the studies reviewed, such as self-efficacy, knowledge, intentions and attitudes have not been strongly supported as correlates [27,75-77]. Self-efficacy has mediated changes in physical activity in several adolescent studies [72,78,79]; however, no studies targeting children have undertaken mediating analysis to confirm mediation pathways.

Of the studies that targeted social environmental potential mediators of children’s physical activity, 37% reported some intervention success with the majority achieving trivial to moderate effect sizes with these variables. Social support, the most commonly targeted potential social environmental mediator in this review, has been identified previously as a consistent correlate of physical activity in children [76,77]. Less than half of the studies reviewed in the current paper showed a positive effect on this potential mediator or a positive effect on the physical activity outcome for studies that targeted this potential mediator. This result may reflect the variation and/or quality of the social support measures used in these studies. A previous review on the validity and reliability of instruments used to assess potential mediators of children’s physical activity reported a lack of appropriate, valid and reliable instruments for measuring constructs such as social support [80], indicating the need to consider the ways in which potential mediators are measured prior to drawing conclusions regarding their use as potential mediators.

The present review also identified a number of studies where the intervention had no significant effect on the potential mediator; however, a significant effect on physical activity was reported. For example, the only study to target change in children’s attraction to physical activity was not successful in effecting such change, but there were significant physical activity outcomes in that study [46]. The mechanism/s through which this change occurred is unclear. The intervention may have achieved its effect on physical activity through another potential mediator or, as discussed above, the measure used to assess the potential mediator may have lacked adequate validity and reliability and was therefore unable to show an effect. Possible reasons for the lack of demonstrated effect on the potential mediators may be due to the wrong mediator being targeted, lack of statistical mediating analysis, lack of power in the sample to detect change, inadequate intervention dose and/or lack of validity and reliability of mediator measures.

In addition to issues regarding instrument reliability and validity, measurement specificity should also be considered. Stathi et al highlight the importance of measuring and reporting the type, intensity and context of physical activity, ensuring the differentiation of the variable constituents of children’s physical activity as activity undertaken for different purposes and intensities is predicted by different correlates and mediated by different variables. It has been suggested that not considering these dimensions of physical activity may result in inaccurate and even misleading estimates of intervention effects [81].

It is important to consider that self-report measures are able to provide estimates of the type, intensity and context of physical activity however the use of these measures is limited due to issues with correlated measurement error when assessing associations and thus biased conclusions [82]. Objective measures also provide limitations as current technology is not able to assess the type and context, particularly at a large scale. However, future research should identify the optimal method of combining self-report and device-based data which may help overcome these issues. Recent methodological advancements in objective physical activity assessment where the use of computer based learning algorithms (For example, artificial neural networks) are being used to estimate activity type may help overcome some of the limitations of objective measures [83,84].

The findings from this review make it difficult to recommend any particular potential mediator as a target for children’s physical activity. This is not to imply that any of the potential mediators reviewed are unimportant, there is simply insufficient evidence that these factors lie on the mediating pathway of children’s physical activity behaviour change. Only one-third of studies reported small to modest changes in the targeted potential mediators, with approximately 75% of these studies reporting positive effects on physical activity outcomes. It is intriguing that a greater number of studies that reported success in changing a potential mediating variable also reported change in children’s physical activity; however, this could also be a reporting bias in the studies. The most frequently targeted potential mediators were cognitive/psychological factors, with only physical activity knowledge having mainly positive outcomes.

### Limitations and strengths

There are limitations to the present review, some of which are due to gaps in the literature itself. Only papers published in the English language were included in this review, and the majority of studies were conducted in the US or Europe. Studies were diverse in character (e.g. mediators targeted and strategies used) and so it was not possible to make recommendations regarding which mediator/s or strategies should be targeted to effect change in physical activity. Several studies may not have been powered to detect significant associations between the intervention and potential mediators; however, effect size calculations were performed for this review to try and aid interpretation of whether results were meaningful.

A quality metric was not applied to this review for several reasons. Inclusion criteria of published studies were deliberately broad so that a more informative representation of the breadth and consistency of potential mediators that children’s physical activity intervention studies have reported could be portrayed. With the scarcity of studies that have systematically reported the targeting of specific mediators, designing strategies that address these mediators or performing statistical mediating analyses, we believe that application of a quality metric to this review would have been pointless given the mediator literature is still so under-developed. The present review was also unable to determine whether studies that targeted specific mediators of change in children’s physical activity applied appropriate strategies to effect these changes. Further, there was such variation between studies in the intervention strategies used it was difficult to draw conclusions about what specifically worked in effective interventions compared to ineffective studies or to link such findings to a match or mis-match between targeted mediators and strategies adopted.

Strengths of the review included the systematic approach adopted and the more inclusive criteria for study inclusion, and the synthesis of evidence of intervention effectiveness on the mediator according to physical activity outcomes.

### Conclusions

Future interventions promoting children’s physical activity should clearly identify and provide a rationale for the theoretical framework used and the hypothesised mediators of change, as well as clearly linking the targeted mediator with the approach used. Potential mediators that target the full ecological framework, in particular the physical, cultural and policy environments, should be tested. Studies outside the US and Europe should be encouraged, and the use of appropriate statistical mediation techniques and valid and reliable measures that are sensitive to change is recommended to test the pathways of behavioural change, thereby informing future intervention development.

## Competing interests

The authors declare they have no conflict of interest to disclose.

## Authors’ contributions

HB and JS conceived the study with input from NP and CH; all of these authors provided intellectual input into the development of the review, data management and discussion. HB carried out the literature search, calculated effect sizes where required, tabulated the results and provided initial draft of manuscript. All authors contributed to interpreting results and reviewing/revising the manuscript.

## Funding

JS is supported by a National Health & Medical Research Council Principal Research Fellowship (APP1026216). CH is funded by a post-doctoral fellowship from the National Heart Foundation of Australia.

## Pre-publication history

The pre-publication history for this paper can be accessed here:

http://www.biomedcentral.com/1471-2458/13/165/prepub

## Supplementary Material

Additional file 1: Table S1 Summary of interventions targeting potential mediators of children’s physical activity. Click here for file
