# Supplementary material for: A systematic review of intervention effects on potential mediators of children’s physical activity
Source: BMC Public Health. 2013 Feb 23;13:165. doi: 10.1186/1471-2458-13-165 (PMC3585884; doi:10.1186/1471-2458-13-165)
Supplement: Additional file 1: Table S1 — Summary of interventions targeting potential mediators of children’s physical activity. [file 1471-2458-13-165-S1.docx]

**Supplementary table. Summary of interventions targeting potential mediators of children’s physical activity.**

| **Author (year)** | **Intervention** | **Setting** | **Strategy**  **(based on information available)** | **Strategies (related to PA)** | **Effect on PA** | **PM and effect of IV on PM** | **Effect size** |
| --- | --- | --- | --- | --- | --- | --- | --- |
| Harrell et al  (2003)  USA  [[1](#_ENREF_1)] | Cardiovascular Health in Children Study (CHIC)  8 week RCT  Theory: SCT & TPB  Sex: B,G  Age: 7 -11 yrs  N: 686 (c), 588 (IV)  No. schools: 33  SES: Low, medium, high  Eth: 74% White, 20% African American | School | Curriculum, PE | Intervention:  Regular classroom teacher provides 2 classes/week on health topics; 3 PE classes/week each including 30 mins PA.  Control:  No contact during IV period. | 0 | Exercise knowledge + | S |
| Bush et al  (1989)  USA  [[2](#_ENREF_2)] | Know Your Body (KYB)  2yr RCT  Theory: SLT  Sex: B, G  Age: Mean age 10.5 yrs (4^th^-6^th^ Grade)  N:431; C: - -; N(IV): - -  No. schools: 9  SES: Low, medium, high  Eth: African American | School | Curriculum,  Family newsletters | Intervention:  2 arms: Gp 1) KYB curriculum (2 x 45 mins module on PA and Fitness+ personalised health screening (results provided) Gp 2) KYB curriculum + health screening (results to parents).  Control:  No KYB curriculum. Parents received health screening results only. | 0 | Health Knowledge + | NC |
| Caballero et al (2003)  USA  [[3](#_ENREF_3)] | Pathways  3 yr RCT  Theory: …  Sex: B, G  Age: Mean age 7.2 yrs (3^rd^–5^th^ Grade)  N(C): 682; N(IV): 727  No. schools: 41  SES: - -  Eth: Native American | School | Curriculum, PE,  Active class breaks, Family newsletters and events | Intervention:  1) Classroom curriculum 2x45 mins/wk for 12 wks;  2) 3x30 min PE sessions per wk + exercise breaks in class time;  3) Family involvement (supportive environments, family events).  Control:  No contact | +/0 | Knowledge +  Self-Efficacy + | S  S |
| Christodoulos et al  (2006)  Greece  [[4](#_ENREF_4)] | 1 yr RCT  Theory: TPB  Sex: B, G  Age: 10-12.5 yrs (6th Grade)  N(C): 49; N(IV): 29  No. schools: 2  SES: - -  Eth: - - | School | Curriculum, PE,  Acitve homework,  Family newsletters, Link to community sport | Intervention:  1) Weekly PE  2) Health education lessons 1/wk (incl. computer  mediated delivery)  3) Homework with family activities  4) Educational material and information about  community based sport programs  5) Parent advice (healthy snacks, encourage PA).  Control: weekly PE, no additional health education | + | PA Intentions +  Attitude + | M    M |
| Edmundson et al  (1996)  USA  [[5](#_ENREF_5)] | Child and Adolescent Trial for Cardiovascular Health (CATCH)  3 yr RCT  Theory: SCT  Sex: B, G  Age: Mean age 8.8 yrs (3^rd^ Grade)  N(C): 2117; N(IV): 2989 (pre-test)  No. schools: 96  SES: - -  Eth: 69% White, 14% Hispanic, 13% African American | School | Curriculum, PE  Family newsletters & events,  Link to community sport | Intervention:  2 arms:  1. Increase PE time >90 mins/wk, intensity MVPA 40%,  health promotion classes consisting of 15, 24 & 16  lessons in Grades 3, 4 & 5, respectively  2. As above + family based component (2 family fun  nights, 19 activity packs over 3 yrs)  Control: Usual curriculum | +/0 | Perceived Support for PA -  Self-Efficacy + | L  T |
| Ernst and Pangrazi  (1999)  USA  [[6](#_ENREF_6)] | Promoting Lifetime Activity in Youth (PLAY)  12 week RCT  Theory: --  Sex: B, G  Age: (4^th^-6^th^ Grade)  N(C): 14 classes; N(IV): 14 classes  No. schools: 5  SES: Low, medium  Eth: Primarily Caucasian & Hispanic | School | Curriculum,  Active class breaks,  Self management, | Intervention:  Daily 15min PA breaks in class-time where teachers taught games and activities for 4 wks. Next 8 wks, instead of PA breaks, children recorded the previous days PA in a recording book, with the goal of achieving 30mins of PA daily after school hrs.  Control:  Daily 15 min breaks in class-time with no prompting to be physically active for 4wks, then recording of time spent watching TV for 8wks | + | Intention: +  Preference +  CAPA (Child attraction to PA) - | M  L  M |
| Goran and Reynolds  (2005)  USA | Interactive Multimedia for Promoting Physical Activity  (IMPACT)  8 week RCT  Theory: SCT  Sex: B, G  Age: 8.8-11.1 yrs (4^th^ Grade)  N(C): 60; N(IV): 62  No schools: 4  SES: - -  Eth: 58% Hispanic | School | Curriculum,  Family newsletters | Intervention:  1) 8 x 45 mins CD-ROM animated lessons  2) 4 x 45 mins classroom lessons  3) 4 x 45 mins family based assignments (12 hrs contact over 8 wks)  Control:  Educational CD-ROMs not relating to health topics | +/0 | PA Beliefs -  Outcome expectancies +  Social norms +  Famil y norms -  Peer norms -  Self-Efficacy + | M  S  T  S  S  T |
| Gortmaker et al  (1999)  USA | Eat Well and Keep Moving  2 yr comparative study with concurrent control  Theory: SCT and BCT  Sex: B, G  Age: 8-10 yrs (4^th^ & 5^th^ Grade)  Cohort: N(C): 289; N(IV): 190.  Cross-sectional: N(C): 180; N(IV): 129.  24hr recall sample: N(C): 162; N(IV): 173  No. schools: 14  SES: Low-medium  Eth: 91% African American | School | Curriculum, PE  Family newsletters and events,  Link to community sport | Intervention:  1) 13 (PA and nutrition) lessons per yr, including 3 PE  lessons (increased MPA and VPA)  2) Campaigns and home activities involving family members promoted in school newsletter and kids projects  3) Linking of parent with community groups.  Control: Usual health education curriculum | 0 | PA Knowledge + | M |
| Marcus et al  (1987)  USA | Know Your Body  (KYB)  2 x 18 Week comparative study with concurrent control  Theory: SLT  Sex: B, G  Age: 9-11yrs (4th & 5th Grade)  N(C): 234; N(IV Gp 1): 688, N(IV Gp 2): 333, N(IV Gp 3): 253 (pre-test)  No. schools: 18  SES: Low  Eth: Mixed | School | Curriculum,  Family newsletters | Intervention:  2 arms: Gp 1) KYB curriculum (2 x 45 mins module on PA and Fitness+ personalised health screening (results provided) Gp 2) KYB curriculum + health screening (results to parents).  Control:  No KYB curriculum. Parents received health screening results only. | + (gp 2)  0 (gp 1) | Fitness knowledge + | NC |
| McKenzie et al  (2004)  USA | Middle-School Physical Activity and Nutrition  (M-SPAN)  2 yr RCT  Theory: SEM  Sex: B, G  Age: 11-14 yrs (6^th^–8^th^ Grade)  N: 1434; N(C): - - ; N(IV): - -  No. schools: 24  SES: 39% low  Eth: 45% non-white | School | Curriculum, PE,  Environment,  Family newsletters,  Community links | Intervention:  Curriculum & environmental/policy:  1) PE program, incl. curriculum material (changed lesson content, structure & teacher behaviour)  2) Environment: increased supervision, equipment & organized activities/promoted in free time  3) Health policy meetings: key school staff met with study team to select environmental policy changes (3x90min meetings/yr, 2-4 policies/yr + action plan)  4) Student health committees (monthly activities - advocacy)  5) Parental education (newsletters, posters, brochures, meetings), 16 articles for newsletter  6) Project team presentations to PTA (11 of 12 boards/schools)  7) $1000 for PE & $2000 PA equipment.  Control: measurement only | + | Enjoyment of PA:  Boys - Girls - | T S |
| Parcel et al  (1989)  USA | Go For Health  2 yr Comparative study with concurrent control  Theory: Organisational change and SLT  Sex: B, G  Age: 3^rd^-4^th^ Grade  N(C): 159; N(IV): 171  No. schools: 4  SES: - -  Eth: Anglo-American 62%, Mexican-American 20%, African American 14% | School | Curriculum, PE | Intervention:  *Go For Health*: 3 program components, 2 related to PA:  1) Curriculum *Children’s Active Physical Education* curriculum 2 semester long units, 6-8 wks each - more time in fitness development  2) Classroom health education: 2x4 wk healthy eating modules, 1x6 wk PA modules.  Control: Usual care | + | Exercise Behavioural Capability +  Self-Efficacy + | NC  NC |
| Cason & Logan  (2006)  USA | Jump Into Foods and Fitness (JIFF)  7 wk Quasi – experimental  Theory: ---  Sex: B, G  Age: 9-11 yrs  N: 130 N(C): 72; N(IV): 58  No. schools: 2  SES: underserved youth  Eth: 71.5% African American | School | Curriculum,,PE,  Family newsletters | Intervention:  1) 7, 1 hour lesson units. Units include information on PA pyramid and PE classes reinforce content  2) Newsletters sent home which are designed to inform parents about program.  Control:  Received IV after intervention completed. | + | Enjoyment + | L |
| Verstraete et al  (2006)  Belgium | SPARK Belgium  2 yr RCT  Theory: SCT  Sex: B, G  Age: 11-12 yrs  N: 764 N(C): 365; N(IV): 399  No. schools: 16  SES: --  Eth: -- | School | Curriculum, PE, Recess activities,  Self management,  After school program | Intervention:  1) Health related PE program and self management program,  2) Classroom based health education lessons, extracurricular PA promotion program (SPARK)  3) Promoted recess PA  4) After school / lunch PA program ran by external PE teacher (voluntary).  Control: usual care | + | Perc. safety -  Enjoyment -  Outcome Expectancies -  Social support -  Self-Efficacy - | T  L  T  T  T |
| Jurg et al  (2006)  Amsterdam | Jump-In  1 year Quasi experimental  Theory: SCT, SEM, TPB, PAPM, SQM, PE and Habit  Sex: B, G  Age: 4th-6^th^ grade  N: 510 N(C): 141; N(IV): 369  No. schools: 6  SES: low  Eth: -- | School | Curriculum,  Homework,  Active Breaks,  Community links,  Family newsletters and events | Intervention:  1) School sports activities – designed to link with club sport  2) Pupil follow up of students sport participation  3) Class moves – regular class breaks  4) Card games (involve assignments to be done in the class and at home)  5) Parental information service – 1 / year  6) Activity week – parents involved.    Control: usual care | + | Awareness -  Outcome Expectancies +  Encouragement -  Social modelling -  Self-Efficacy -  Intention -  Habit + | NC  NC  NC  NC  NC  NC  NC |
| Palmer et al  (2005)  USA | Cross over design  Theory: --  Sex: B, G  Age: 5^th^ Grade  N(C): 82 (cross-over); IV: 90  No. schools: 8  SES: Low-medium  Eth: Predominantly Caucasian | School | Curriculum | Intervention:  Web-based program/module ‘Healthy Hearts 4 Kids’ 2x≤50mins/wk. Units on: cardiovascular function, PA, nutrition, tobacco (information, guidelines, quizzes, writing activities to reinforce content, PA & nutrition habits, feedback).  Control: cross over | 0 | Knowledge -  Attitude - | S  T |
| Rowland et al  (2003)  UK | 1 yr RCT  Theory: --  Sex: B, G  Age: 2nd & 5th Grade  N(C): 672; N(IV): 714  No. schools: 21  SES: - -  Eth: - - | School | Curriculum,  Family newsletters,  Environment | Intervention:  16 hrs of expert assistance from a school travel coordinator to develop & implement travel plans  Control: Received £150 in compensation for their time | 0 | Parental Concerns about travel to school - | T |
| Manios  (2002)  Greece | 6 year RCT  Theory: --  Sex: B, G  Age: 1^st^ – 6^th^ grade  N(C): 444 N(IV): 602  No. schools: 40  SES: --  Eth: -- | School | Curriculum, PE,  Active breaks,  Active transport campaigns,  Family newsletters | Intervention:  1) Classroom PA lessons  2) Short PA breaks during lessons,  3) 2 active commuting to school campaigns  4) Activity box (equipment)  5) Parent fact sheets  6) PE teacher PD  7) Computer tailoring program.  Control: usual care | + | Knowledge + | M |
| Harrison et al  (2006)  Ireland | Switch Off Get Active  16 week RCT  Theory: --  Sex: B, G  Age: mean: 10.2  N(C): ; N(IV):  No. schools: 9  SES: social disadvantage  Eth: -- | School | Curriculum,  Self management | Intervention:  10 lesson, teacher lead IV based school lessons and accompanying activity modification tasks – workbooks and diaries to record PA / screen time.  Control: Usual classes | + | Self-Efficacy + | T |
| Bergh et al  (2012)  Norway | Health in Adolescents  (HEIA)  20 month RCT  Theory: --  Sex: B, G  Age: Mean: 11 yrs  N(C):510 N(IV): 908  No. schools: 37  SES: --  Eth: -- | School | Curriculum, PE,  Active breaks,  Active transport,  Accessibility,  Family newsletters | Intervention:  1) 1 x 90 min class concerning PA behaviour in relation to energy balance  2) Short PA breaks during lessons  3) 2 x active commuting to school campaigns  4) Activity box – sport and play equipment for recess  5) 3 PA fact sheets for parents  6) 1 x Professional Development session for PE teachers  Control: usual care | +/0 | Enjoyment +  Perceived Social Support +  Self-Efficacy - | M  S  T |
| Sallis et al  (1997)  USA | SPARK  (Sports, Play, and Active Recreation for Kids)  Quasi experimental  Theory: --  Sex: B, G  Age: 4^th^ grade  N(C): 360 N(IV teacher):331 N(IV specialist): 360  No. schools: 7  SES: --  Eth: 82% European American | School | Curriculum, PE,  Homework,  Family information,  Self management | 3 arms:  1) Specialist led (certified PE specialist),  2) Teacher led (classroom),  3) Control  Intervention:  Aim- increase PA during PE classes and outside school  1) Classes designed to promote high levels of PA, be enjoyable and teach movement skills (3 days a week)  2) Self-management program also taught in 30 minute classroom sessions  3) Homework and monthly newsletters for home.  Control: usual PE taken by untrained classroom teachers | +/0 | Self-perception -  Family support -  Enjoyment -  Intention - | T  S  S  S |
| Baranowski et al  (1990)  USA | Community gym  14 week RCT  Theory: --  Sex: B, G  Age: Mean age 10.6-10.9 yrs (5^th^–7^th^ Grade)  N(C): 52; N(IV): 59  SES: range  Eth: African-American | Family | Education, PA,  Family involvement in activities / counselling | Intervention:  1) 1x90min education session/wk (behavioral counselling, goal setting, small group education for 7 wks  2) 1x30min MPA session/wk.  Control: no contact during IV period | - | PA knowledge -  Self-efficacy - | NC  NC |
| Baranowski et al  (2003)  USA | Baylor GEMS Pilot study  12 weeks RCT  Theory: SCT  Sex: G  Age: 8 yrs  N(C): 16; N(IV): 19  SES: - -  Eth: African-American | Family | Education, PA,  Camp  Self management | Intervention:  4 wk Summer Day Camp, then 8 wk Internet IV for girls & for parents (weekly): social support buddies, parental modelling, PA skills & exposure, pedometer self-monitoring.  Control:  Generic 4-wk day camp, followed by generic 8 wk internet program | 0 | PA preference - | L |
| Beech et al  (2003)  USA | Memphis GEMS pilot study  12 week RCT  Theory: SCT  Sex: G  Age: 8-10 yrs  N(C): 18; N(IV): 21  SES: - -  Eth: African-American | Family | Education, PA,  Family newsletters | Intervention:  Knowledge, behaviour change skills & dance or aerobics: Interactive weekly sessions with 1) girls and 2) parents  Control:  3x90min sessions over 12 wks (self-esteem) | 0 | Self-efficacy (PA) -  Self-concept -  Outcome expectancies -  Preference - | S  S  S  S |
| Robinson et Al  (2003)  USA | Stanford GEMS pilot study  12 week RCT  Theory: SCT  Sex: G  Age: 8-10 yrs  N(C): 33; N(IV): 28  SES: Low  Eth: African-American | Family | Education, PA,  Family newsletters | Intervention:  After-school dance classes 5 days/wk at community centres & 5-lesson home visiting program with families to reduce SB  Control:  Newsletters and lectures on diet & PA | 0 | Enjoyment -  Self-esteem - | S  M |
| Story et al  (2003)  USA | Minnesota GEMS Pilot study  12 week RCT  Theory: SCT  Sex: G  Age: 8-10 yrs  N(C): 28; N(IV): 26  SES: Low  Eth: African-American | Family | After school curriculum and PA,  Family newsletters and events | Intervention:  2x1 hr after-school sessions/wk; weekly family packs; 2 family nights; 1 motivational telephone call; tailored letter in wk 7  Control:  3 meetings over 12 wks (arts/crafts & self-esteem activities) | + | PA self concept -  PA preference -  Outcome expectancies -  Self-efficacy -  Parental support -  PA home environment - | S  S  S  M  S  M |
| Rhodes et al  (2010)  Canada | 4 week RCT  Theory: --  Sex: G, B  Age: 4-10 yrs  N(IV + planning): 31; N(IV): 34  SES: --  Eth: -- | Family | Education,  Family newsletters | Intervention:  Group 1– IV only, Group 2- IV + planning  Families sent the Canadian family PA guidelines, Active Living recreation guide. Group 2 families also received planning material (workbook and calendar) – asked to read instructions and follow for 4 weeks. | 0 | Intention -  Perceived Behavioural Control - | NC  NC |
| Pate et al  USA | Active Winners  18 month  Comparative study with concurrent control  Theory: SCT and Pender’s Health Promotion Model  Sex: B, G  Age: Mean age 11 yrs (5^th^ Grade)  N(C): 224; N(IV): 148  No. schools: 6  SES: Low  Eth: primarily African-American | Community | After school PA,  Family newsletters,  Community links | Intervention:  4 main components:  1) Active Kids: intensive summer & after-school PA  program;  2) Active Home: newsletters for families;  3) Active School: formation of committees to improve  school environment;  4) Active Community: newspaper articles and PA at local Events  Control:  No intervention | 0 | PA beliefs  Boys - Girls -  Social influences  Boys + Girls -  PA intentions  Boys + Girls - | S S  T M  T M |
| Trost et al  (2009)  USA | 4 week RCT  Theory: --  Sex: B, G  Age: 105 child parent dyads  N(C): 40 child parent dyads;  N(IV): 65 child parent dyads  SES: --  Eth: -- | Family | Curriculum, PA,  Family events | Intervention:  PA curriculum module (4 lessons) during regular Sunday school classes  + family activities designed to promote PA at home (3 activities)  Control:  Normal Sunday School program | 0 | Parental support  Father -  Parental Support Mother + | T  L |
| Kelder  (2005)  USA | Coordinated Approach to Child health  CATCH Kids Club  Quasi experimental  Theory: SCT  Sex: B, G  Age: Mean 9 yrs (Grades K-5)  N: 69  No. schools: 16  SES: --  Eth: 43% White, 34% Hispanic | After school | After school PA | Intervention:  1) 5 module education component (teacher led after school),  2) PA component (activity box containing fun active games and activities)  Control:  Usual after school care | + | Self-efficacy - | L |
| Annesi et al  (2007)  USA | ‘Youth Fit For Life’  12 week Quasi-experimental  Theory: --  Sex: B, G  Age: mean 10.1 yrs  N(AS): 128 ; N(PE): 113  SES: --  Eth: African American | After school | After school PA,  Self management  PE,  Self management | 2 groups:  1) After school group – 3 day/week afterschool care - games, resistance training, self-management program (workbook included)  2) PE group - 2 day/week PE format, delivered by PE specialists. 2 sessions/week, 45 mins a session. Same content as 1^st^ group, but delivered in a gymnasium. | + | Group 1:  Physical Self Concept +  Self-Efficacy -  Group 2:  Physical Self Concept -  Self-Efficacy + | L  S  L    S |
| Chen et al  (2009)  USA | Active Balanced Childhood (ABC)  RCT (wait list control)  Theory: SCT  Sex: B, G  Age: 8-10 yrs  N(C): 32 ; N(IV): 35  SES: --  Eth: Chinese American | Family | Education, PA,  Family newsletters and events | Intervention:  1) Small group family sessions – local community  2) Children participate in PA sessions (parents involved in 2)  3) Provided with Pedometers, activity diary, books about PA  4) Family component – workshop, workbook and video  Control:  Wait-listed | + | Knowledge +  Self-efficacy - | M  T |

**Key:**

Effect on PA: + = significant effect on physical activity (PA) outcome, 0= no significant effect on PA outcome, +/0= mixed result

PM and effect of IV on PM: PM = potential mediator, + = positive significant effect on PM, - = null effect on PM

Effect size: T= trivial (<0.2), S= small (>0.2-0.5), M= moderate (>0.5-0.8), L= large (>0.8), NC=not able to be calculated.

1. Harrell, J.S., et al., *Effects of a school-based intervention to reduce cardiovascular disease risk factors in elementary-school children: The Cardiovascular Health in Children (CHIC) Study.* The Journal of Pediatrics, 1996. **128**(6): p. 797-805.

2. Bush, P.J., et al., *Cardiovascular risk factor prevention in Black school children: the "Know Your Body" evaluation project.* Health Education Quarterly, 1989. **16**(2): p. 215-227.

3. Caballero, B., et al., *Pathways: a school-based, randomized controlled trial for the prevention of obesity in American Indian schoolchildren.* Am J Clin Nutr, 2003. **78**: p. 1030-8.

4. Christodoulos, A.D., et al., *Attitudes towards exercise and physical activity behaviours in Greek schoolchildren after a year long health education intervention.* British Journal of Sports Medicine, 2006. **40**: p. 367-371.

5. Edmundson, E., et al., *The effects of the Child and Adolescent Trial for Cardiovascular Health upon psychosocial determinants of diet and physical activity behavior.* Preventive Medicine, 1996. **25**(4): p. 442-454.

6. Ernst MP and Pangrazi RP, *Effects of a physical activity program on children's activity levels and attraction to physical activity.* Pediatr Exerc Sci, 1999. **11**: p. 393-405.
